# Application of sirolimus in an infant presenting with a life-threatening lymphatic malformation of the head and neck: a case report

**DOI:** 10.3389/fped.2025.1587330

**Published:** 2025-04-24

**Authors:** Shuying Yu, Xiaowen Guo

**Affiliations:** ^1^ Department of Pharmacy, Hangzhou Children’s Hospital, Hangzhou, China; ^2^ Department of Clinical Pharmacy, Xinhua Hospital Affiliated to Shanghai JiaoTong University School of Medicine, Shanghai, China

**Keywords:** lymphatic malformation, sirolimus, rapamycin, infant, individualized treatment

## Abstract

Extensive lymphatic malformations in the head and neck region pose a significant risk due to potential airway compression, and conventional treatment modalities have proven largely ineffective. Currently, systemic administration of sirolimus is recognized as a promising therapeutic approach for complex lymphatic malformations. Nevertheless, the appropriate dosage, optimal blood concentration, efficacy, and safety profile of sirolimus in pediatric patients remain inadequately characterized. In this report, we present a clinical case involving a 3-month-old male infant diagnosed with tongue lymphatic malformation, treated with sirolimus. It is noteworthy that the trough concentration of sirolimus is influenced by both genetic and non-genetic factors, including physiological and pathological conditions, as well as drug-food and drug-drug interactions in pediatric patients. Despite the sirolimus concentration below the target range during treatment, a reduction in tumor size was observed. Additionally, based on the patient's medical history, adjustments in medication, and liver function assessments, the pharmacist has excluded the likelihood of sirolimus-induced hepatotoxicity. This case underscores the safety and efficacy of sirolimus in managing extensive lymphatic malformations of the head and neck in infants. Regular monitoring and analysis of variations in sirolimus blood concentrations, coupled with long-term follow-up observations, are essential for enhancing treatment efficacy and minimizing toxicity risks.

## Introduction

Lymphatic malformation (LM), also referred to as lymphangioma, is a prevalent congenital vascular malformation that infrequently resolves spontaneously (1, 2). It can result in disfigurement, malformation, and potentially life-threatening compression of critical organs, such as the airway (3, 4). The treatment strategy is contingent upon the lesion's location, extent, and depth. Conventional treatment modalities primarily encompass surgical resection, sclerotherapy injection, and laser therapy, although therapeutic outcomes can vary significantly among individuals (5–7).

Pharmacological intervention has emerged as a focal point in contemporary research on LM management. Given the correlation between LMs and PIK3CA mutations in somatic cells, which activate the PI3K/AKT/mTOR signaling pathway involved in lymphangiogenesis and angiogenesis (8), the mTOR pathway inhibitor sirolimus (rapamycin) has gained widespread application in recent years for the treatment of severe LMs (9). The mechanistic target of sirolimus (mTOR) facilitates cellular growth, proliferation, and lymphangiogenesis by modulating the phosphorylation of downstream targets, specifically p70S6 kinase and eIF4E-binding protein 1, via the mTORC1 and mTORC2 complexes (10). Sirolimus exerts its effects by binding to intracellular FK binding proteins, forming complexes that subsequently associate with mTORC1, thereby disrupting its capacity to transmit signals to downstream effectors. This interruption inhibits protein synthesis and subsequent cellular proliferation and angiogenesis, ultimately impeding the progression of lymphatic malformations. Currently, there is an absence of standardized guidelines regarding the dosage, optimal blood concentrations, treatment duration, efficacy, and safety of sirolimus in pediatric patients, as evidenced by retrospective case reports and case series available in the literature (9, 11). Furthermore, notable disparities exist in the pharmacokinetic parameters between pediatric and adult populations (12). Consequently, significant individual variability persists in the “off-label” use of sirolimus for treating LMs in pediatric patients.

In this context, we present our clinical experience with the administration of sirolimus in infants diagnosed with lingual LM.

## Case description

A 3-month-old male patient was admitted to the Department of Pediatric Intensive Care Medicine at Xinhua Hospital, affiliated with Shanghai Jiaotong University. The patient presented with a history of tracheotomy status and recurrent ventilatory dysfunction persisting for seven days, alongside a diagnosis of lingual LM for three months. He was delivered at 37 weeks and 4 days gestation via cesarean section, with a birth weight of 3,600 grams and an Apgar score of 10. At birth, the patient exhibited no cyanosis but was crying. On physical examination, the patient demonstrated persistent tongue protrusion with bilateral cystic swelling in the cheek and neck regions. MRI confirmed the diagnosis of LM. The patient was immediately transferred to the neonatal intensive care unit (NICU) and underwent endotracheal intubation. Subsequently, on the 6th day, as well as during the 1st and 2nd months postnatally, polidocanol was administered via injection into both cheeks, while bleomycin was injected into the tongue. One month thereafter, a tracheotomy was conducted, accompanied by intralingual injection of polidocanol, and a metal sheath was positioned at the site of incision. However, due to the inadequate stability of the sleeve, the infant experienced recurrent ventilation difficulties.

Upon admission (Day −0), a physical examination revealed the presence of cystic masses located at the anterior and lateral regions of the neck. The patient weighed 4,400 grams with a body surface area of 0.25 m^2^. On the fifth day of admission (Day −5), a tracheoplasty was conducted, followed by a near-total resection of cervical and submandibular LMs, along with a local injection of bleomycin. Postoperative facial swelling was observed, and a CT scan revealed that the LM was exerting pressure on the airway. Given that the patient's primary condition continued to progress and surgical intervention was not indicated at that time, a multidisciplinary consultation was held on Day −13. Subsequently, adjuvant therapy with sirolimus oral solution was initiated at a dose of 0.2 mg once daily QD, calculated based on body surface area. Furthermore, to prevent pneumocystis pneumonia during sirolimus therapy, an oral regimen of trimethoprim-sulfamethoxazole (TMP-SMZ, comprising 400 mg of sulfamethoxazole and 80 mg of trimethoprim per tablet) was prescribed at a dosage of 30 mg/kg/day, administered twice daily, three times per week.

One week after medication (Day −22), first TDM result showed a Cminss of 3.37 ng/ml, which did not achieve target trough levels within the range of 10–15 ng/ml. Sirolimus dose was increased to 0.4 mg QD. After one week (Day −30), the retest level of sirolimus was even lower (only 1.64 ng/ml) with mildly elevated liver enzymes. Alanine aminotransferase (ALT) and aspartate aminotransferase (AST) are 32 U/L and 89.6 U/L, respectively. During this period (from Day −27 to Day −33), the patient experienced diarrhea, with 5 bowel movements per day. Considering the reduction of facial swelling in the patient (Day −33), we continued to maintain the current dosage. About two weeks later TDM (Day −39) still showed low Cminss of 2.13 ng/ml, meanwhile ALT and AST increased to 389.0 U/L and 741.3 U/L, respectively. Since it cannot be ruled out that sirolimus may cause liver damage, according to the sirolimus instructions, when liver damage occurs, the maintenance dose can be reduced by about 1/3–1/2, and then adjusted to 0.3 mg QD, while also administering liver protective medications: Ursodeoxycholic acid capsules and injectable glutathione. The genetic testing of the patient suggested that the genotype of CYP3A4 * 1B (A392G) was AA, and the genotype of CYP3A5 *3 (A6986G) was GG. TMP-SMZ has been discontinued due to stock shortage on Day −35. The patient's blood routine examination showed low hemoglobin levels (Hb < 90 g/L) from Day −18 to 41.

**Figure 1:**
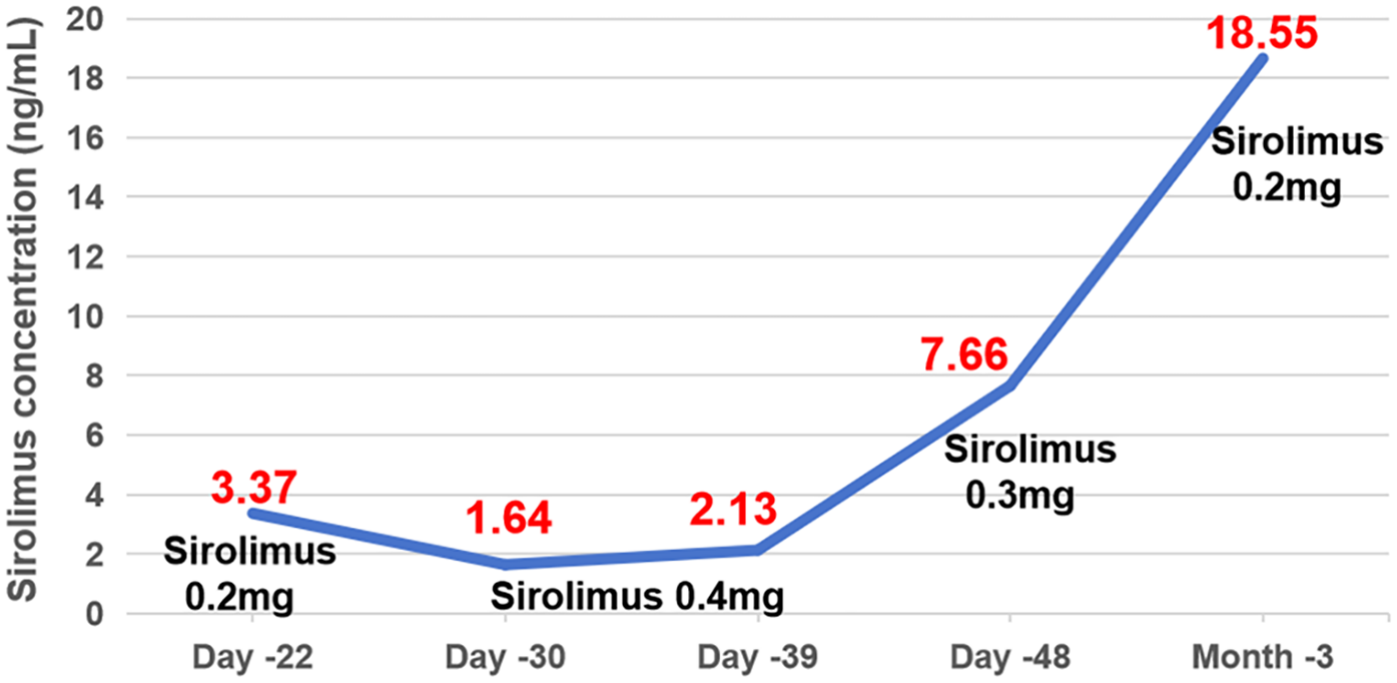
The individualized dosing regimen of sirolimus and the evolution of Cminss values.

**Figure 2:**
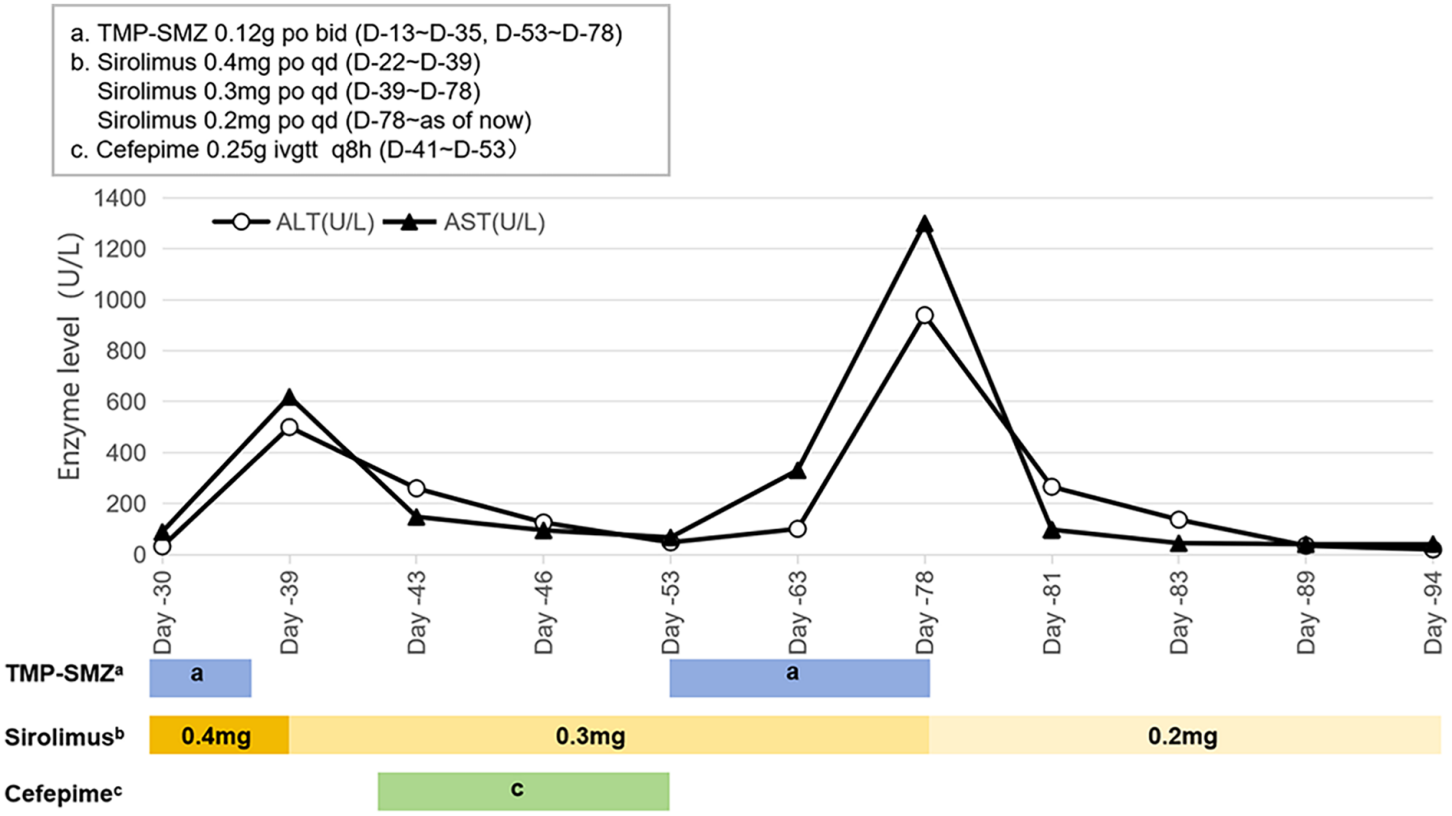
Dynamic changes in the drug treatment regimen and the liver function indicators.

On Day −41, the patient's body temperature rose to 38.7 °C, C-reactive protein (CRP) level of 86 mg/L, procalcitonin (PCT) of 1.66 ng/ml, trachea aspirate culture, chest-x-ray and CT demonstrated *carbapenem-resistant Acinetobacter baumannii* (CRAB) pneumonia, and cefepime was added for anti-infective treatment. On Day −48, the plastic surgery consultation concluded that the patient's current tumor size has been reduced by about one-third, which was more effective. Additionally, Cminss of sirolimus rose to 7.66 ng/ml. Five days later (Day −53), ALT and AST significantly decreased to 48 U/L and 67.8 U/L. Chest x-ray showed significant improvement in pneumonia. Cefepime was stopped, while liver-protective medications and TMP-SMZ continued to be used due to resumption of supply. After 10 days of retesting (Day −63), the levels of liver enzymes ALT and AST increased to 101.0 and 331.3 U/L, respectively. No special treatment was given, and follow-up visits were conducted at the discharge clinic. On Day −78, the patient's outpatient monitoring showed a significant increased in liver enzymes (ALT: 939 U/L, AST: 1,300 U/L), therefore we discontinued TMP-SMZ and reduced sirolimus to 0.2 mg QD for administration. After one week, the liver function returned to normal and Cminss of sirolimus was 18.55 ng/ml (Figures 1, 2).

## Discussion

The patient in question had undergone numerous sclerosing agent injections and localized interventions, including surgical procedures, since birth, yet the outcomes had been suboptimal. Computed tomography (CT) imaging revealed that the tumor continued to exhibit diffuse infiltrative growth, compromising the airway and respiratory function. Current research suggests that for extensive or refractory LM, the oral administration of sirolimus, with informed consent from the patient's family, may be considered to reduce the lesion size. This approach could facilitate subsequent definitive surgical interventions or enhance the efficacy of traditional treatments such as sclerotherapy, potentially leading to a complete cure (13, 14). Consequently, based on a comprehensive review of the literature, the administration of sirolimus is deemed an appropriate therapeutic strategy for the patient in this case.

Currently, there is ongoing debate regarding the appropriate dosage of oral sirolimus for the treatment of LMs. In comparison to the 2016 edition of the Diagnosis and Treatment Guidelines for Hemangiomas and Vascular Malformations published by the Chinese Society for the Study of Vascular Anomalies (CSSVA) (15), the 2019 edition has incorporated the initial dosage (0.1 mg/kg/day or 0.8 mg/m^2^/day) and therapeutic blood concentration range (10–15 ng/ml) of sirolimus for the management of LM in pediatric patients (16). A retrospective study involving 105 patients, ranging from newborns to 17-year-old children, who were treated with sirolimus for head and neck lymphangiopathy, found that the majority received an initial dose of 0.8 mg/m^2^/12 h, while a minority received alternative dosages such as 0.8 mg/(m^2^·day) or 0.08 mg/(kg·day) (5). Another clinical study investigating the use of sirolimus for neonatal airway LMs indicated that an initial dose of 0.8 mg/m^2^/12 h could lead to adverse reactions due to elevated monitoring trough concentrations. Consequently, for infants aged 6 weeks to 6 months, a dosage of 0.8 mg/(m^2^·day) was suggested (17). Furthermore, a quantitative pharmacology-based modeling approach determined an accurate initial dosing regimen for sirolimus tailored to the age of newborns and infants with complex vascular abnormalities. For infants aged 3–4 months, the initial dosing required to achieve a target blood concentration of 10–15 ng/ml was 1.4 mg/(m^2^·day), whereas a concentration of 5–10 ng/ml necessitated 0.9 mg/(m^2^·day) (12). Based on this evidence, to mitigate the risk of rapid onset adverse reactions, the pharmacist recommended to administer a lower initial dose of 0.2 mg once daily (0.8 mg/m^2^/day), calculated based on body surface area, with subsequent adjustments made according to the target blood trough concentration.

Given the narrow therapeutic window and significant interindividual variability of sirolimus, its therapeutic efficacy, as well as the occurrence and severity of adverse reactions, are closely linked to blood drug concentration. Therefore, it is imperative to conduct blood drug concentration monitoring one week post-initiation of therapy. In this case, the patient's initial steady-state trough concentration was 3.37 ng/ml, which falls short of the guideline-recommended target trough concentration range of 10–15 ng/ml for the treatment of LM. According to the dosage adjustment method outlined in the manual, the new dosage can be calculated using the formula: new dosage = current dosage × (target blood drug concentration current blood drug concentration), suggesting a theoretical increase to 0.6 mg/day. Researchers established an accurate administration protocol for sirolimus in pediatric patients with vascular abnormalities through the application of Monte Carlo simulations grounded in mathematical modeling. The findings indicated that the optimal sirolimus dosage for children aged 3 weeks to 2 years ranged from 0.7 to 1.6 mg/(m^2^·day) (equivalent to 0.175–0.4 mg/day), achieving the desired therapeutic concentration (18). Based on the dosing guidelines provided in the literature, pharmacists recommend a gradual titration of the dosage, initiating treatment at 0.4 mg once daily. Given the extended half-life of sirolimus, it is advised to reassess the blood concentration levels at least 7–14 days following any dosage adjustment.

Notably, despite a twofold increase in the sirolimus dose, the blood concentration levels recorded on Days −30 and −39 were observed to be lower than the initial measurements. Various factors influence the blood concentration of sirolimus, including genetic determinants (such as CYP3A4/CYP3A5/ABCB1 genotypes) and non-genetic factors (such as physiological and pathological conditions, drug-food and drug-drug interactions, and administration methods) (19, 20). Sirolimus is primarily metabolized by the hepatic enzymes CYP3A4 and CYP3A5, with single nucleotide polymorphisms at these gene loci significantly influencing its biotransformation. Studies indicate that the mutation rate of the CYP3A4 (392A>G, rs2740574) gene is nearly 0% among the Chinese population (21), whereas the CYP3A5 *3 (6986A>G, rs776746) mutation occurs at a frequency of 71%–76% (22). Consequently, the impact on sirolimus metabolism in this population is predominantly associated with CYP3A5. Genetic analysis of this patient revealed homozygosity for the CYP3A5 *3 allele (GG, *3/*3 mutation). In a study by Zhang et al. (21), involving healthy subjects administered oral sirolimus, it was demonstrated that individuals carrying the CYP3A5 *3/*3 genotype exhibited significantly increased *in vivo* exposure to sirolimus, as measured by AUC_0–144_ and C_max_, compared to those with the CYP3A5 *1/*1 (wild-type homozygous) or CYP3A5 *1/*3 (mutant heterozygous) genotypes, indicating a marked reduction in enzyme activity. Further research involving Chinese kidney transplant recipients showed that patients with the *3/*3 genotype had higher stable trough concentrations and dose requirements for sirolimus compared to those with CYP3A5 *1/*1 and *1/*3 genotypes (23), suggesting a slower metabolic rate for sirolimus in these individuals. In theory, administering conventional or reduced doses of sirolimus may achieve the target blood drug concentration; however, this does not necessarily correspond to the patient's actual blood drug concentration. Regarding non-genetic factors, sirolimus interacts primarily with certain foods and drugs, notably grapefruit juice and CYP3A4 and P-gp inducers or inhibitors, like rifampicin, voriconazole, erythromycin. Nevertheless, no food or drug was identified with a clinically significant interaction in pediatric drug use. Given that sirolimus is predominantly excreted via the fecal biliary route, the patient's diarrhea from Day −27 to Day −33 could have resulted in increased excretion and a subsequent reduction in blood drug concentration. Concurrently, the patient's complete blood count from Day −18 to Day −41 indicated anemia, and it is important to note that sirolimus is primarily distributed in whole blood red blood cells rather than plasma. It is hypothesized that anemia may lead to an increased concentration of sirolimus in free plasma, thereby enhancing its hepatic metabolism. Conversely, there exists a negative correlation between hematocrit levels and the apparent clearance rate of sirolimus (24), suggesting that anemia could elevate its clearance rate and consequently reduce the whole blood concentration of the drug. In conclusion, the child's diarrhea and anemic condition are likely contributing factors to the observed low blood drug concentration. Nevertheless, given the observed reduction in facial swelling at the current dosage, which indicates clinical efficacy, the pharmacist advises maintaining the current medication dosage. Should the blood drug concentration remain low after a one-week follow-up, an increase in dosage is recommended.

On Day 39, the child's liver enzyme levels were elevated and subsequently demonstrated a continuous upward trend. The Child-Pugh score was calculated to be 7 points, comprising bilirubin (1 point), albumin (2 points), prothrombin time (2 points), ascites (1 point), and hepatic encephalopathy (1 point). According to the guidelines, it is advised to decrease the maintenance dose of sirolimus by one-quarter (i.e., 0.3 mg daily), and subsequently reassess the blood concentration to observe any significant increase from baseline levels. The pharmacist evaluated the underlying factors as follows: (1) The child did not experience further episodes of diarrhea or anemia during this period. (2) Clinical studies demonstrated that, in comparison to healthy individuals, the clearance rates of orally administered sirolimus are reduced by 31.8%, 36.0%, and 67% in patients with Child-Pugh A (5–6 points), B (7–9 points), and C (10–15 points) liver dysfunction, respectively (25). The child had Child-Pugh B, moderately impaired hepatic function, which resulted in decreased clearance of sirolimus, leading to drug accumulation and elevated blood concentrations. (3) Additionally, literature indicated that inflammatory cytokines in children with acute lymphoblastic leukemia can significantly suppress the expression and activity of CYP enzymes during infections, causing a sudden increase in sirolimus blood levels (26). In this particular case, the patient recently experienced aspiration and pulmonary infection following the dislodgement of the tracheostomy tube. Consequently, the observed pathological and physiological conditions may account for the abrupt elevation in the patient's blood concentration of sirolimus.

A systematic review of 20 studies concerning the treatment of LMs with sirolimus revealed that the target trough concentration was predominantly maintained within the ranges of 10–15 ng/ml or 5–15 ng/ml (5). In a study by Margolin et al. (27), sirolimus was administered to a child with diffuse LM, achieving trough concentration levels predominantly below 10 ng/ml, and occasionally below 2 ng/ml. This regimen resulted in a significant reduction in tumor volume and improvements in swallowing and speech function. Another clinical investigation employed low target trough concentration levels (4–10 ng/ml) of sirolimus in the treatment of 12 cases of refractory LM, demonstrated a clinical response rate comparable to studies utilizing higher target trough concentrations (10–15 ng/ml), while also enhancing drug tolerance (28). By the 20th day of sirolimus treatment, the patient's facial swelling had diminished. By the 34th day, a consultation with a plastic surgeon confirmed that the patient's tumor had reduced by approximately one-third, suggesting that a lower trough concentration of sirolimus (<10 ng/ml) was also efficacious in the treatment of LM. Despite not achieving the guideline-recommended target trough concentration range for LM treatment (10–15 ng/ml), the pharmacist advised maintaining the current therapeutic regimen.

Presently, the adverse reactions associated with sirolimus in LM treatment are considered mild and manageable. The most common side effects in children include oral mucosal ulcers, liver dysfunction (elevated transaminase levels), and elevated blood lipids, all of which are dose-dependent (29). Abnormalities in AST levels are positively correlated with the blood concentration of sirolimus (30). During sirolimus treatment, the child exhibited elevated liver enzyme levels, with infection and primary disease influences on liver function being ruled out. The initial hypothesis was that the liver injury was attributable to either sirolimus or TMP-SMZ. Drug-induced liver injury in pediatric patients is typically diagnosed through exclusion and lacks specific diagnostic criteria. The systematic evaluation, based on the RUCAM causality assessment scale (31), combined with the absence of liver enzyme abnormalities at elevated sirolimus concentrations, suggested a high likelihood (>8 points) that the liver injury is associated with TMP-SMZ.

While current guidelines advocate for the administration of sirolimus in conjunction with oral TMP-SMZ to prevent Pneumocystis carinii infection in infants and young children, multicenter retrospective studies indicated that the incidence of LM infection remained low with long-term sirolimus monotherapy. Prophylactic use of TMP-SMZ may be advantageous for patients at elevated risk of infection, such as those with malignant tumors or those concurrently receiving other immunosuppressants (32). However, for patients not at high risk, the potential adverse reactions associated with TMP-SMZ warrant careful consideration. Therefore, we recommend a judicious approach in the selection of TMP-SMZ for anti-infective treatment.

The immunosuppressive properties of sirolimus may result in severe infectious complications in pediatric patients undergoing treatment. Two infant patients with Kaposi-type vascular endothelial tumors, who were subjected to prolonged sirolimus therapy, developed cough and respiratory distress during the course of treatment, ultimately succumbing to pulmonary infections (33). A multicenter retrospective study conducted in Europe indicated that serious adverse events, such as respiratory infections and sepsis, predominantly occurred within the first year of sirolimus treatment in children with vascular abnormalities. Specifically, 47% of these events were reported within the first three months, 35% between three to twelve months, and only 18% after more than one year of treatment (34). These findings underscore the importance for clinicians and caregivers to maintain heightened vigilance for infectious complications throughout the duration of sirolimus therapy. It is recommended that a thorough assessment of the patient's condition be conducted prior to initiating sirolimus treatment, carefully weighing the therapeutic benefits against the potential risk of infection. During the course of treatment, individualized dosage adjustments are imperative, accompanied by vigilant monitoring for signs of infection and regular assessment of infection markers. Prompt pathogen testing is essential to facilitate targeted supportive therapy. It is crucial to adhere to scheduled vaccinations for infants and young children, while abstaining from the administration of live vaccines. Additionally, it is recommended to minimize contact with infected individuals and rigorously enforce hand hygiene and disinfection protocols to prevent infection.

Currently, there is no consensus on the optimal duration of sirolimus treatment for LM, necessitating further clinical evidence from case applications. According to Hammill et al. (35), the average onset time for sirolimus treatment was 25 days; however, the time required to achieve the optimal therapeutic effect remains uncertain. Retrospective studies indicate that the treatment duration for pediatric head and neck LMs typically ranged from 6 months to 4 years (5). Consequently, we advocate for individualized treatment plans, with the duration determined by factors such as the type, size, location, growth trend, and associated symptoms of the malformation.

## Patient perspective

The neonate was diagnosed with glossal LM during the initial physical examination at birth and was promptly transferred to the NICU via endotracheal intubation. Despite multiple administrations of sclerosants and surgical interventions, the outcomes were suboptimal, culminating in acute respiratory failure due to airway compression. After careful consideration of available options, a decision was made to initiate an off-label treatment regimen with sirolimus. Although the use of this pharmacological agent for pediatric LM remains limited, we resolved to collaborate closely with medical professionals and pharmacists to explore this novel therapeutic approach. Throughout the treatment process, the healthcare team provided comprehensive information regarding the potential risks and precautions associated with the medication, and we adhered strictly to their guidance, vigilantly monitoring for any adverse reactions. Although the child experienced hepatic function abnormalities during the treatment, timely medical intervention facilitated recovery, and the tumor exhibited continued regression. We express our profound gratitude to the medical team for their dedicated care and treatment, as well as for granting informed consent for the publication of this case.

## In conclusion

The “off-label” use of sirolimus presents an effective and safe therapeutic option for pediatric patients with complex and life-threatening head and neck LMs. Nevertheless, the narrow therapeutic window of sirolimus results in significant individual variability, particularly among pediatric patients. The pharmacist evaluated the patient's blood drug concentration monitoring results from genetic and pharmacokinetic perspectives and collaborated with the clinical physician to adjust the medication regimen. Concurrently, adverse reactions observed during treatment were investigated to identify their causes, and efforts were made to actively collaborate with clinical experts to optimize the treatment protocol. It is important to note that due to the limited sample size and short follow-up duration in individual cases, the long-term efficacy and safety of sirolimus remain undetermined. Further prospective clinical trials are necessary to establish the appropriate dosage, target blood concentration, and assess the long-term efficacy of sirolimus in treating pediatric head and neck LM. The effective diagnosis and management of lymphatic malformation in this 3-month-old Chinese male infant offers valuable insights for developing personalized therapeutic strategies in similar pediatric cases.

## Data Availability

The raw data supporting the conclusions of this article will be made available by the authors, without undue reservation.
